# What Happens during Natural Protein Fibre Dissolution in Ionic Liquids

**DOI:** 10.3390/ma7096158

**Published:** 2014-08-28

**Authors:** Jingyu Chen, Kylie Vongsanga, Xungai Wang, Nolene Byrne

**Affiliations:** Institute for Frontier Materials, Deakin University, Waurn Ponds, VIC 3216, Australia; E-Mails: jingyu.chen@deakin.edu.au (J.C.); k.vongsanga@deakin.edu.au (K.V.); xungai.wang@deakin.edu.au (X.W.)

**Keywords:** protein, ionic liquid (IL), dissolution, hydrogen bond, disulphide bond

## Abstract

Here, we monitor the dissolution of several natural protein fibres such as wool, human hair and silk, in various ionic liquids (ILs). The dissolution of protein-based materials using ILs is an emerging area exploring the production of new materials from waste products. Wool is a keratin fibre, which is extensively used in the textiles industry and as a result has considerable amounts of waste produced each year. Wool, along with human hair, has a unique morphology whereby the outer layer, the cuticle, is heavily cross linked with disulphide bonds, whereas silk does not have this outer layer. Here we show how ILs dissolve natural protein fibres and how the mechanism of dissolution is directly related to the structure and morphology of the wool fibre.

## 1. Introduction

The utilization of natural protein fibres as renewable alternatives to current petroleum-based polymers is an area of growing potential. Wool and silk are among the most commonly used natural animal fibres in the world [1].

**Figure 1:**
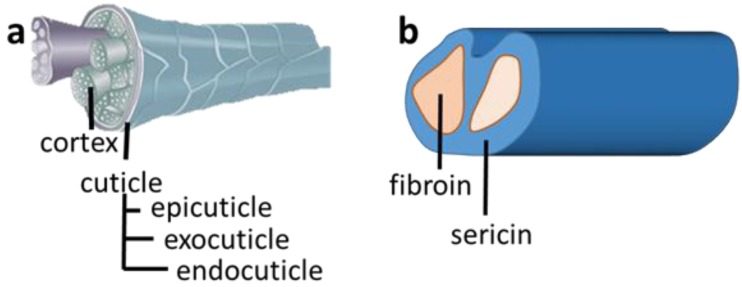
Schematic illustration of the structure of: (**a**) wool fibre [8]; and (**b**) silk fibre.

Wool is a keratin-based biopolymer used extensively in the textile industry [2,3,4]. Wool, like several other keratin-based fibres including llama, cashmere and human hair all share a similar but unique structure [5]. A wool fibre consists of two distinct regions (Figure 1a), the sulphur-rich outer layer known as the cuticle and the inner component called the cortex [2]. The cuticle contains numerous disulphide bonds which connects the peptide chains and is composed of three layers, the epicuticle, exocuticle and endocuticle. Of the three layers, the exocuticle has the highest sulfur content [2]. The cuticle acts like a protective layer and imparts properties such as wettability, tactile properties and is responsible for the felting of wool [2]. The cortex, making up to 90% of the mass of a fibre, is comprised of cortical cells, which are bounded together with each other and with the cuticle cells by a cell membrane complex (CMC) [2].

On the other hand silk fibres obtained from the cocoons of silkworm are mainly composed of two protein monofilaments [6], as shown in Figure 1b. The structural protein component termed the fibroin and this is covered by another protein, sericin [6]. The sericin, is a water soluble protein with glue-like properties. The sericin is often removed to give silk fibres a high lustre and silk feeling [6]. Both wool and silk fibres exhibit crystalline structures, with the polypeptide chains folded into specific conformations, such as α-helix for wool, and β-sheets, for silk [2,6,7]. These conformations are stabilised by inter- and intra-molecular bonds respectively.

Traditional methods for the dissolution of wool usually employ multiple chemical steps since the cuticle layer which contains the disulphide bonds are generally either oxidized or reduced during dissolution. Under oxidative methods, the disulfide bonds are turned into cysteic acid (–CH_2_–SO_3_H) with oxidants, such as cuprammonium hydroxide [9], or H_2_O_2_ [10]. In the reduction methods, the disulphide bonds are reduced to –SH by chemicals such as sodium bisulphite [11], 2-mercaptoethanol [12,13], and thioglycolic acid (TGA) [14]. Many of these methods also employ urea to disrupt the hydrogen bonding between the peptide chains within the cortex region [11].

To dissolve the degummed fibres of silk, disruption of the bridging bonds between the polypeptide chains of the fibroin is required. Solvents such as aqueous inorganic salt solutions [15], fluorinated organic solvents [16] and concentrated acids [17] are normally used, because of their strong ability to disrupt hydrogen bonding [18].

In recent times, ionic liquids (ILs) for the dissolution of natural fibres have been extensively studied [19,20,21,22,23,24,25]. The dissolution of natural protein fibres using ILs represent a new avenue for IL processing which can include the development of new materials and the selective extraction of protein for tissue engineering [26,27,28].

In this manuscript, we observe the dissolution of natural protein fibres in different ILs using optical microscopy in real time. In comparison to the dissolution of cellulosic material in ILs, a lesser number of publications exist with respect to the use of ILs to dissolve protein fibres [23,29,30]. To date, ILs which have been used to dissolve protein fibres are those based on the imidazolium cation with either a chloride or acetate anion [24,31,32,33,34]. They have been selected predominately due to their ability to dissolve cellulose based on hydrogen bond breaking capabilities. While both cellulosic fibres and protein fibres can be classified as natural fibres, the unique morphology of wool sets it apart from even silk, which does not contain the same high content of disulphide bonds.

We have studied a range of ILs which has been used to dissolve either wool or cellulosic materials in previous publications [21,22,24,30,35,36]. We find that in wool and human hair, the cortex dissolves preferentially and after prolonged time the cuticle dissolves, in an inside-out dissolution mechanism. We compared this to silk and show that silk dissolves progressively from the surface, from the outside to inside.

## 2. Results and Discussion

### 2.1. Dissolution Process

**Figure 2:**
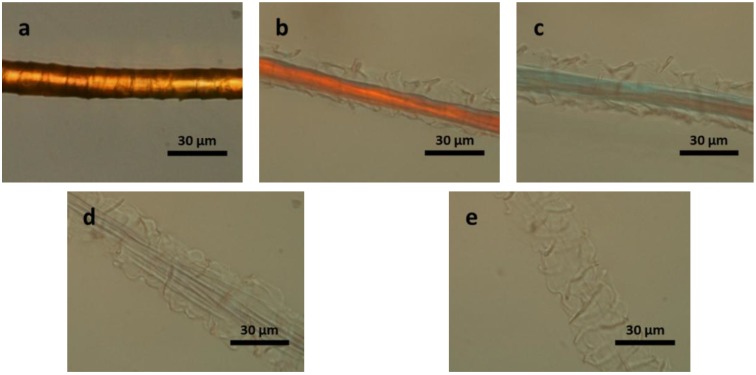
Polarizing optical microscope (POM) images of a wool fibre in [Bmim]OAc at 120 °C: (**a**) original wool fibre; for (**b**) 5 s; (**c**) 50 s; (**d**) 100 s; and (**e**) 180 s.

The *in situ* dissolution of a single wool fibre in the select IL was observed using polarized optical microscopy. The sequence of dissolution of wool fibre in [Bmim]OAc at 120 °C is shown in Figure 2a–e. It can be seen that initially the cuticle swells (Figure 2a) followed by swelling of the cortex (Figure 2b). Then the crystallinity in the cortex is being destroyed as the cortex becomes transparent, suggesting that dissolution is occurring first in the cortex (Figure 2c,d). Figure 2e shows that the cortex completely dissolves leaving behind the cuticle. The time taken for the dissolution of the cortex was 3 min, while time taken to completely dissolve the cuticle was significantly longer, more than 1 h (Figure S1 in Supplementary). The extended time required to dissolve the cuticle is likely linked to the high content of disulphide bonds within the cuticle. The swelling of the wool fibre during dissolution suggests that the cortex may remain inside the fibre even in the dissolved state.

**Figure 3:**
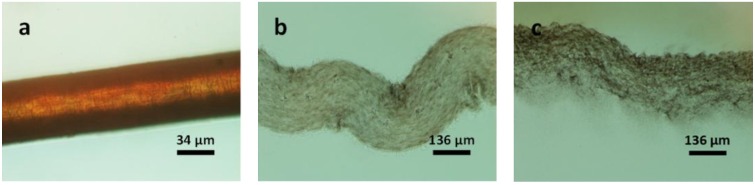
POM images of a black hair fibre in [Bmim]OAc at 120 °C: (**a**) original fibre; for (**b**) 25 min; and (**c**) 90 min.

To observe this, we have applied the single fibre *in situ* observation technique, to darkly pigmented hair. The structure of hair is similar to wool in that the outer cuticle layer contains the disulphide bonds while the inner cortex is better represented by hydrogen bonds. By using a dark fibre, the coloured pigments within the cortex can be observed. Figure 3a,b shows the dissolution process of a dark hair strand, indeed the cortex remains inside the casing of the cuticle even in the dissolved state. During dissolution, the hair fibre expands, swelling four times its original diameter as highlighted by the scale bar in the image below. After 90 min, the cuticle starts to dissolve and the coloured pigments disperse from the broken place throughout the IL solution as shown in Figure 3c.

### 2.2. Solubility of Wool in Various Ionic Liquids

Next we wanted to observe if the dissolution process of wool fibre were similar amongst other ILs. Table 1 shows the ILs tested for dissolution, the time taken and an indication of whether swelling of the cuticle and cortex occurred. We have observed a range of wool and cellulose dissolving ILs [21,22,24,30]. We find in all ILs tested the mechanism for dissolution is similar, that is cuticle swelling, cortex swelling and dissolution followed by cuticle dissolution. Table 1 list the ILs in order from fastest to slowest time taken to completely dissolve the wool fibre. [Bmim]OAc is the most effective solvent [Choline]TGA is also an effective solvent (Figure S2 in Supplementary), [Bmim]CI is not as effective when compared to the [Choline]TGA or [Choline]Pn. We also tried the protic [TMG]Pn as this IL has been shown to dissolve cellulose; however, dissolution was not achieved: the cortex which is the crystalline component and containing majority of the hydrogen bonds was not dissolved. Indeed for wool the order of best solvent to worst solvent does not follow that recently found for cellulose suggesting the nature of the cuticle and the hydrogen bonds makes it more complex and effective basicity may not be the best measure to determine effective wool dissolving solvents [29]. The temperature used for the [TMG]Pn was reduced to 100 °C due to the protic nature of this IL. Even at this temperature, after 3 h we found that the IL was no longer a true 1:1 mixture with base having been lost (as determine from nuclear magnetic resonance (NMR), showing a 30% loss of base). As previously reported [30], we also found that temperature was an important parameter for dissolution. When the temperature for dissolution in [Bmim]OAc was lowered by 20–100 °C, the time taken for dissolution increased by an order of magnitude and the time taken to dissolve the cortex was 14 min as opposed to 3 min at 120 °C.

**Table 1:** Observed dissolution times and temperatures of single wool fibre tests in various ionic liquids (ILs).

IL	Temperature (°C)	Time (min)	Cuticle swollen	Cortex dissolved
[Bmim]OAc	120	3	yes	yes
[Choline]TGA	120	10	yes	yes
[Choline]Pn	120	45	yes	yes
[Bmim]Cl	120	90	yes	yes
[TMG]Pn	100	390	yes	no

### 2.3. The Disulphide Bonds in the Cuticle

Given the importance of the disulphide bonds in the cuticle, we sought to explore what effect dissolution with ILs would have when the disulphide bonds where chemically reduced or oxidized, using TGA [37], or hydrogen peroxide (H_2_O_2_) [38].

**Figure 4:**
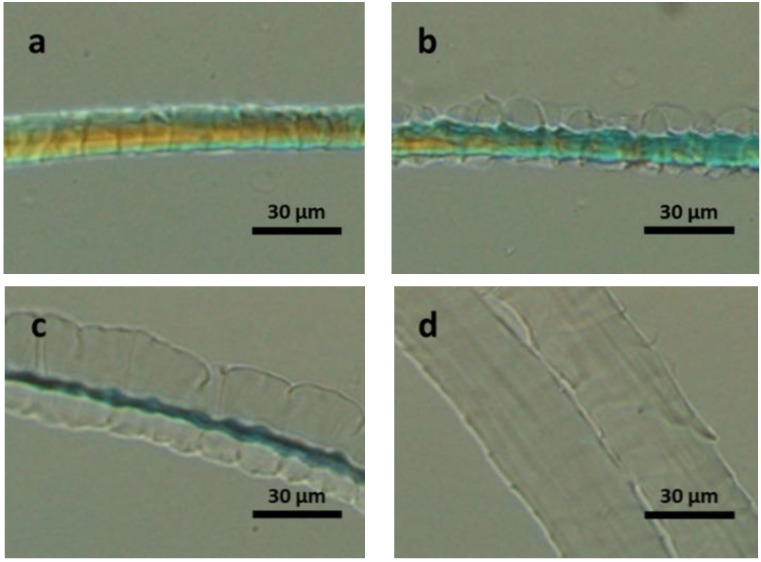
POM images of thioglycolic acid (TGA) pretreated wool fibre dissolving in [Bmim]OAc at 120 °C for: (**a**) 0 s; (**b**) 5 s; (**c**) 15 s; and (**d**) 30 s.

After treatment with TGA, partial reduction of the disulfide bonds are achieved, resulting in the formation of –SH (Figure S3a in Supplementary). When compared to native wool dissolution, wool treated with TGA dissolved five times faster [Bmim]OAc at 120 °C (Figure 4). The overall process, that is: cuticle swelling, cortex swelling, cortex dissolution and finally cuticle dissolution was found to be the same.

**Figure 5:**
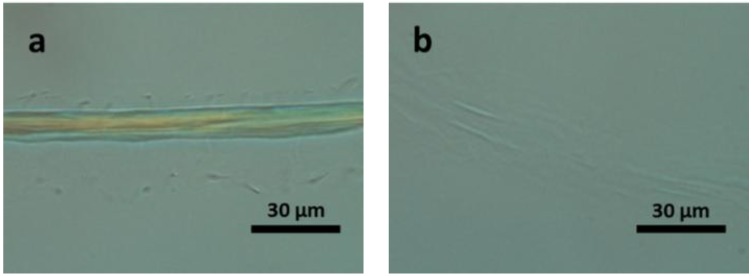
POM images of hydrogen peroxide (H_2_O_2_) pretreated wool fibre dissolving in [Bmim]OAc at 120 °C for: (**a**) 5 s; and (**b**) 180 s.

Next oxidized the wool surface using H_2_O_2_ [10,39]. This oxidized fibre exhibited the most dramatic response to the IL (Figure 5) with the cuticle fully swelling in 5 s, compared with 100 s for the native fibre (Figure 2). Total dissolution was achieved in 180 s using [Bmim]OAc. Interestingly here both the cuticle and the cortex dissolved simultaneously.

Furthermore, an Allwörden reaction [40,41] type phenomena was observed (*i.e.*, the observation of “bubbling” of the cuticle) during the dissolution of TGA pretreated wool fibres. As shown in Figure 1, the cuticle is made up of three layers: epicuticle, exocuticle, and endocuticle. The bubbles observed suggest that with minimised hindrance of disulphide bonds in the exocuticle, the materials underlying epicuticle dissolved immediately when in contact with the IL. However, on the samples pretreated with H_2_O_2_, we have observed no bubbles and the cuticle and cortex dissolved simultaneously. This indicates that the treatment with TGA does not damage the epicuticle membrane, while pretreatment with H_2_O_2_ does. Based on this, the technique of dissolution of single fibre in ILs exhibits potential application in evaluating the degree of damage to wool fibre after processing.

### 2.4. Dissolution of Silk Fibre in Ionic Liquids

**Figure 6:**
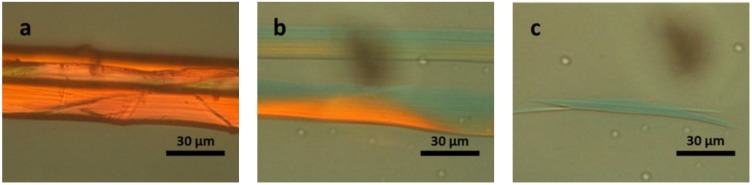
POM image of silk fibres dissolving in [Bmim]OAc at 120 °C: (**a**) native silk fibre; after (**b**) 120 s; and (**c**) 240 s.

Finally, we observed the dissolution of domestic silk fibre using [Bmim]OAc. The complete dissolution of a single silk fibre took approximately 4 min in [Bmim]OAc at 120 °C, which was similar to wool dissolution. Nonetheless in contrast to wool, no obvious swelling was seen during dissolution, instead the diameter of the fibre reduced as dissolution proceeded, indicating that dissolution proceeded from the surface (Figure 6).

## 3. Experimental Section

### 3.1. Materials

1,1,3,3-Tetramethylguanidine (99%), propionic acid (≥99.5%), TGA (≥99.5%), choline hydroxide (46 wt%, aqueous) and 1-butyl-3-methylimidazolium chloride ([Bmim]Cl), and 1-butyl-3-methylimidazolium acetate([Bmim]OAc) both 98% were purchased from Sigma-Aldrich (Castle Hill, Australia) and used as received, without further purification. However, the purchased ILs were dried prior to use. Water content of the ILs was determined by 899 Karl Fisher Coulometer (Metrohm, Herisau, Switzerland), and was found to be in the range of 0.8%–2%. 1,1,3,3-Tetramethylguanidinium propionate [TMG]Pn and choline propionate ([Choline]Pn), choline thioglycolate ([Choline]TGA) were prepared according to the literature methods [21,42]. Merino wool, 19.5 µm was a gift from Australian Wool Innovations (Sydney, Australia). Silk cocoons (bombyx mori) were purchased from Shanghai, China. They were degummed in water at 120 °C for 1 h, and dried at 55 °C overnight. Virgin black hair from an Asian female were washed with detergent, rinsed in water, and dried at 55 °C before using.

### 3.2. Thioglycolic Acid Pre-Treatment of Wool

Wool fibres were treated with TGA according to a previous paper [37]. Wool (0.50 g) was immersed in an aqueous solution of TGA (30 mL, 6 wt%, pH = 9, adjusted with NH_4_OH) at room temperature. After 15 min, the fibres were collected and washed with water (300 mL) in triplicate and then dried at 55 °C overnight.

### 3.3. Hydrogen Peroxide Pre-Treatment of Wool

The process was following a reported literature method [38]. H_2_O_2_ (9 mL, 30 wt% aqueous solution) was mixed with deionized water (300 mL) and NH_4_OH was added until a pH of 8 was achieved. Wool (0.10 g) was immersed in the solution for 6 h at 50 °C, with occasional stirring. The fibres were collected and washed with water (300 mL) in triplicate prior to drying at 55 °C overnight.

### 3.4. Characterization

Infrared spectra were recorded with a Bruker LUMOS FTIR Microscope (Billerica, MA, USA) in ATR mode, with accumulation of 64 scans at 4 cm^−1^ resolution.

Raman measurements were conducted using a Renishaw InVia Raman Microspectrometer (Renishaw, Gloucestershire, UK) with diode laser at 785 nm. Spectra were recorded by scanning the 300–1800 cm^−1^ region with 4 × 10 s accumulation scans at 50% energy. Normalization of Raman spectra was carried out based on the C–H band around 1450 cm^−1^, since the peak was not influenced by the chemical treatment [43].

**Figure 7:**
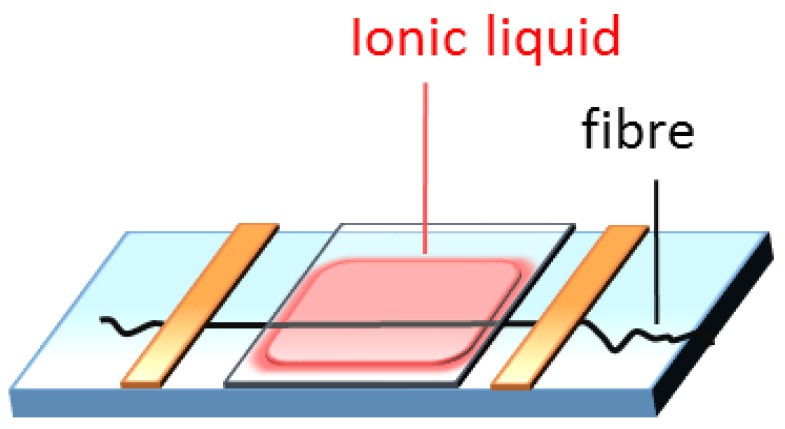
Schematic illustration of the sandwich structure for observation of a single wool fibre dissolution under POM.

Fibres were gently stretched to straighten and fixed on a glass slide with tape, and then heated on the hot stage of the microscope. IL was dropped on the glass slide and a glass coverslip was carefully applied as shown in Figure 7.

The process of dissolution of a single fibre was recorded with Nicon 80i Elipse Polarizing Optical Microscope (POM, Melville, NY, USA) equipped with camera and hot stage. Images were processed with free software ImageJ.

## 4. Conclusions

Here, we have visually studied the dissolution process of several natural protein fibres in various ILs. For wool fibres and human hairs, the dissolution process was observed to proceed with cuticle swelling. This followed IL penetration into the cortex, leading to further swelling and finally complete dissolution of the cortex. The rate of dissolution was shown to have great variation among ILs and was obviously influenced by the temperature of dissolution. Pretreated wool samples via oxidative and reductive methods supported the theory of the S–S bonds, found in the cuticle which makes it more difficult to dissolve in comparison with the cortex. In contrast, the dissolution of silk proceeds from the surface where no swelling was observed, which also supports the theory regarding the S–S bonds as silk lacks such bonds.

## Supplementary Materials

Supplementary materials can be accessed at: http://www.mdpi.com/1996-1944/7/9/6158/s1.

## References

[1] Animal Fiber—Wikipedia, the Free Encyclopedia. http://en.wikipedia.org/wiki/Animal_fiber.

[2] Lewis D.M., Rippon J.A. (2013). The Coloration of Wool and other Keratin Fibres.

[3] Feughelman M. (1997). Mechanical Properties and Structure of Alpha-Keratin Fibres: Wool, Human Hair and Related Fibres.

[4] Wallenberger F.T., Weston N.E. (2004). Natural Fibers, Plastics and Composites.

[5] Marshall R.C., Orwin D.F.G., Gillespie J.M. (1991). Structure and biochemistry of mammalian hard keratin. Electron Microsc. Rev..

[6] John M.J., Thomas S., Chemistry R.S.O. (2012). Natural Polymers: Composites.

[7] Simpson W.S., Crawshaw G.H. (2002). Wool: Science and Technology.

[8] About the Fibre—The Woolmark Company. http://www.woolmark.com/learn-about-wool/about-the-fibre.

[9] Kelly R.J., Worth G.H., Roddick-Lanzilotta A.D., Rankin D.A., Ellis G.D., Mesman P.J.R., Summers C.G., Singleton D.J. (2006). Production of Soluble Keratin Derivaties. U.S. Patent.

[10] Timmons S.F., Blanchard C.R., Smith R.A. (2000). Porous and Bulk Keratin Bio-Polymers. U.S. Patent.

[11] Lees K., Peryman R.V., Elsworth F.F. (1954). The solubility of wool in urea-bisulphite solution and its use as a measure of fibre modification. J. Soc. Dyers Colour..

[12] Yamauchi K., Yamauchi A., Kusunoki T., Kohda A., Konishi Y. (1996). Preparation of stable aqueous solution of keratins, and physiochemical and biodegradational properties of films. J. Biomed. Mater. Res..

[13] Odonnell I.J., Thompson E.O. (1964). Studies on reduced wool 0.4. Isolation of major component. Aust. J. Biol. Sci..

[14] Blanchard C.R., Smith R.A., Timmons S.F. (2000). Method of Making and Cross-Linking Keratin-Based Films and Sheets. U.S. Patent.

[15] Sashina E.S., Bochek A.M., Novoselov N.P., Kirichenko D.A. (2006). Structure and solubility of natural silk fibroin. Russ. J. Appl. Chem..

[16] Yao J., Masuda H., Zhao C., Asakura T. (2001). Artificial spinning and characterization of silk fiber from bombyx mori silk fibroin in hexafluoroacetone hydrate. Macromolecules.

[17] Ha S.-W., Tonelli A.E., Hudson S.M. (2005). Structural studies of bombyx mori silk fibroin during regeneration from solutions and wet fiber spinning. Biomacromolecules.

[18] Wang Q., Chen Q., Yang Y., Shao Z. (2012). Effect of various dissolution systems on the molecular weight of regenerated silk fibroin. Biomacromolecules.

[19] Goujon N., Wang X., Rajkowa R., Byrne N. (2012). Regenerated silk fibroin using protic ionic liquids solvents: Towards an all-ionic-liquid process for producing silk with tunable properties. Chem. Commun..

[20] Swatloski R.P., Spear S.K., Holbrey J.D., Rogers R.D. (2002). Dissolution of cellose with ionic liquids. J. Am. Chem. Soc..

[21] King A.W.T., Asikkala J., Mutikainen I., Järvi P., Kilpeläinen I. (2011). Distillable acid–base conjugate ionic liquids for cellulose dissolution and processing. Angew. Chem. Int. Ed..

[22] Idris A., Vijayaraghavan R., Rana U.A., Fredericks D., Patti A.F., MacFarlane D.R. (2013). Dissolution of feather keratin in ionic liquids. Green Chem..

[23] Silva R., Wang X., Byrne N. (2013). Tri-component bio-composite materials prepared using an eco-friendly processing route. Cellulose.

[24] Xie H., Li S., Zhang S. (2005). Ionic liquids as novel solvents for the dissolution and blending of wool keratin fibers. Green Chem..

[25] Brandt A., Ray M.J., To T.Q., Leak D.J., Murphy R.J., Welton T. (2011). Ionic liquid pretreatment of lignocellulosic biomass with ionic liquid-water mixtures. Green Chem..

[26] Furth M.E., Atala A., van Dyke M.E. (2007). Smart biomaterials design for tissue engineering and regenerative medicine. Biomaterials.

[27] Reichl S. (2009). Films based on human hair keratin as substrates for cell culture and tissue engineering. Biomaterials.

[28] Reichl S., Borrelli M., Geerling G. (2011). Keratin films for ocular surface reconstruction. Biomaterials.

[29] Hauru L.K., Hummel M., King A.W., Kilpelainen I., Sixta H. (2012). Role of solvent parameters in the regeneration of cellulose from ionic liquid solutions. Biomacromolecules.

[30] Parviainen A., King A.W., Mutikainen I., Hummel M., Selg C., Hauru L.K., Sixta H., Kilpelainen I. (2013). Predicting cellulose solvating capabilities of acid-base conjugate ionic liquids. ChemSusChem.

[31] Idris A., Vijayaraghavan R., Rana U.A., Patti A.F., MacFarlane D.R. (2014). Dissolution and regeneration of wool keratin in ionic liquids. Green Chem..

[32] Hameed N., Guo Q.P. (2009). Natural wool/cellulose acetate blends regenerated from the ionic liquid 1-butyl-3-methylimidazolium chloride. Carbohydr. Polym..

[33] Li R., Wang D. (2013). Preparation of regenerated wool keratin films from wool keratin-ionic liquid solutions. J. Appl. Polym. Sci..

[34] Lovejoy K.S., Lou A.J., Davis L.E., Sanchez T.C., Iyer S., Corley C.A., Wilkes J.S., Feller R.K., Fox D.T., Koppisch A.T. (2012). Single-pot extraction-analysis of dyed wool fibers with ionic liquids. Anal. Chem..

[35] Wang H., Gurau G., Rogers R.D. (2012). Ionic liquid processing of cellulose. Chem. Soc. Rev..

[36] Meng Z.J., Zheng X.J., Tang K.Y., Liu J., Qin S.F. (2012). Dissolution of natural polymers in ionic liquids: A review. E-Polymers.

[37] Kuzuhara A., Hori T. (2013). Analysis of heterogeneous reaction between reducing agents and keratin fibers using Raman spectroscopy and microspectrophotometry. J. Mol. Struct..

[38] Hogg L.J., Edwards H.G.M., Farwell D.W., Peters A.T. (1994). FT Raman spectroscopic studies of wool. J. Soc. Dyers Colour..

[39] Alter H., Bit-Alkhas M. (1969). Infrared analysis of oxidized keratin. Text. Res. J..

[40] Fraser R.D.B., Rogers G.E. (1955). The bromine allwörde reaction. Biochim. Biophys. Acta.

[41] Rippon J.A., Lewis D.M. (1992). The structure of wool. Wool Dyeing.

[42] Muhammad N., Man Z., Bustam M., Mutalib M.I.A., Wilfred C., Rafiq S. (2011). Dissolution and delignification of bamboo biomass using amino acid-based ionic liquid. Appl. Biochem. Biotechnol..

[43] Kuzuhara A. (2013). Analysis of internal structure changes in black human hair keratin fibers resulting from bleaching treatments using Raman spectroscopy. J. Mol. Struct..

