# Supplementary material for: What Happens during Natural Protein Fibre Dissolution in Ionic Liquids
Source: Materials (Basel). 2014 Aug 28;7(9):6158–68. doi: 10.3390/ma7096158 (PMC5456163; doi:10.3390/ma7096158)

Supplementary Materials

Figure S1. Polarizing optical microscope (POM) image of wool dissolution in a vial in [BMIM]Ac at 120 °C for 2 h with stirring. Remaining of cuticle can be seen clearly.

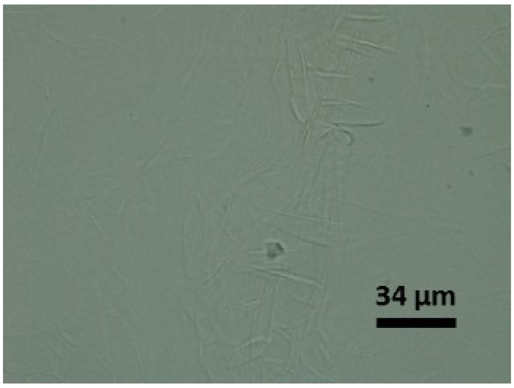

Figure S2. POM image of wool fibre dissolved in [Choline]TGA at 120 °C for 10 min. TGA: thioglycolic acid.

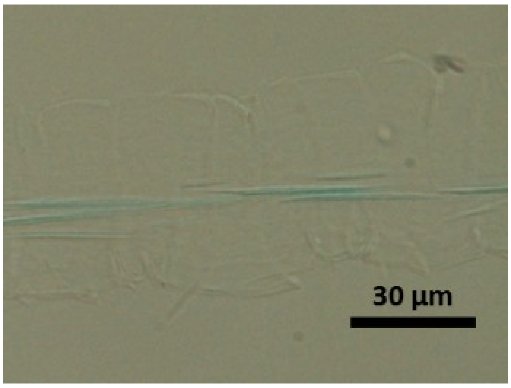

Figure S3. (**a**) Raman spectra of wool fibres treated in aqueous solution of TGA; and (**b**) Fourier transform infrared spectroscopy (FTIR) spectra of wool fibres treated in aqueous solution of hydrogen peroxide (H_2O_2).

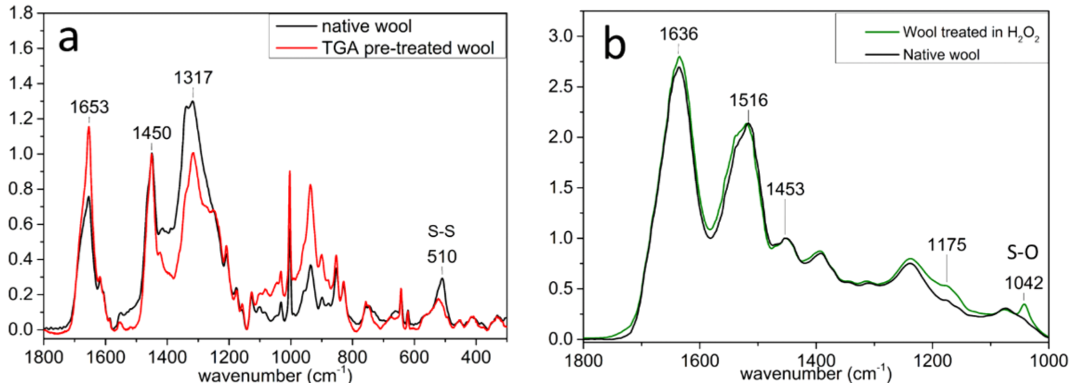

Supplement: Supplementary file 1 [file materials-07-06158-s001.pdf]
